# Suicide due to fear of COVID-19, in the last month of pregnancy, leads to neonatal seizure: A case report

**DOI:** 10.1016/j.amsu.2021.103119

**Published:** 2021-11-24

**Authors:** Javad Boskabadi, Saeed Kargar-Soleiman abad, Shahrokh Mehrpisheh, Elham Pishavar, Roya Farhadi

**Affiliations:** a Department of Clinical Pharmacy, Faculty of Pharmacy, Mazandaran University of Medical Sciences, Sari, Iran; b Student Research Committee, Faculty of Medicine, Mazandaran University of Medical Sciences, Sari, Iran; c Department of Neonatology, Faculty of Medicine, Mazandaran University of Medical Sciences, Sari, Iran; d Department of Pharmaceutical, Biotechnology, School of Pharmacy, Mashhad University of Medical Sciences, Mashhad, Iran; e Department of Neonatology, Pediatrics Infectious Diseases Research Center, Mazandaran University of Medical Sciences, Sari, Iran

**Keywords:** COVID-19, Pregnancy, Mental health, Neonatal seizure, Case report

## Abstract

**Introduction:**

and importance: Limited data are available about various effects of COVID-19 on pregnancy. On the other hand, the COVID-19 pandemic could exacerbate anxiety or schizophrenia symptoms.

**Case presentation:**

The patient is a 5-day-old newborn, whom his mother suffers from schizophrenia, depression and anxiety disorders. The young pregnant mother gets delusions of being infected with Covid-19, thus attempts suicide with Sertraline, Clonazepam, Quetiapine and Rispeirdone, although she was in the last week of pregnancy. The newborn baby referred to our neonatal ward with seizure and apnea. Phenytoin and caffeine were administered leading to some degree of symptom relief, but due to the dermatologic reactions of phenytoin, they were replaced with levetiracetam.

**Clinical discussion:**

The Covid-19 may increase levels of anxiety and depression or exacerbation of schizophrenia symptoms, especially in pregnant women suffering from mental disorders. In addition, there are evidence supporting the occurrence of neonatal malformations as a result of exposure to antipsychotic drugs during the first trimester of pregnancy.

**Conclusion:**

Investigating the role of antidepressant and antipsychotic drugs in the perinatal period, especially near delivery has received less attention so far; thus further studies are required to determine the safety of these drugs.

## Highlights

• The COVID-19 pandemic could exacerbate anxiety or schizophrenia symptoms.
• The pregnancy may be associated with a fear of COVID-19.
• Antipsychotic drugs may cause neonatal malformations.

## 1 Introduction

The coronavirus disease 2019 (COVID-19) was first reported in December 2019 in Hubei Province, China [1].Although all ages and genders are at risk for COVID-19 infection, limited information exists on various effects of COVID-19 on pregnant women [2]. According to reports, pregnant patients have often been excluded from the clinical trials, and most of the studies on COVID-19 have been conducted on non-pregnant population [3].

Research has shown that pregnancy is associated with a fear of COVID-19 infection [4]. The pregnancy period is frequently accompanied by mental problems, especially in women with previously diagnosed psychiatric and anxiety disorders [5]. High stress of COVID-19 pandemic and news of its spread, financial stress on individuals who have lost their jobs, death caused by the virus, disruption in health services and treatment process, and limitation of referral to medical centers are all factors that could contribute to the occurrence and excavation of mental health disorders. All the above-mentioned reasons lead to the emergence of new psychotic disorders in individuals with no previous history of mental health disorders and can exacerbate the psychotic symptoms of COVID-19 patients [6].

Psychotic disorders have harmful outcomes such as suicide, homicide, and other dangerous behaviors. Based on this insight, the suicide rate is usually introduced as an index for assessment of mental health in the general population. Overall, 90% of suicide cases are associated with at least one mental health disorder such as psychosis or depression [7,8].

Pregnant women suffering from mental diseases are more susceptible to suicide compared to the general population [9]. To the best of our knowledge, none of the studies so far have focused on the safety of antipsychotic drugs and antidepressants in the last month of pregnancy and near delivery period.

Given the significance of the effect of Covid-19 on mental health, the purpose of this report was to draw attention to assessment of mental health in the Covid-19 pandemic and call for further investigations in this regard.

## 2 Case presentation

This study complies with the Declaration of Helsinki ethical standards, and corresponds with key components of CARE guidelines and methodology[10]. A 5-day-old newborn boy with a birth weight of 3300 g presented with seizure, frequent apnea, increased blood ammonia and lactate, and apparent meconium aspiration as the chief complaint. He was referred from the birth center to the neonatal intensive care unit. His mother was suffering from schizophrenia, major depression, and anxiety disorder and was on risperidone, quetiapine, sertraline, and clonazepam medication. Except for the baby's mother, there was no history of psychiatric disorders in other first-degree relatives.

**Fig. 1:**
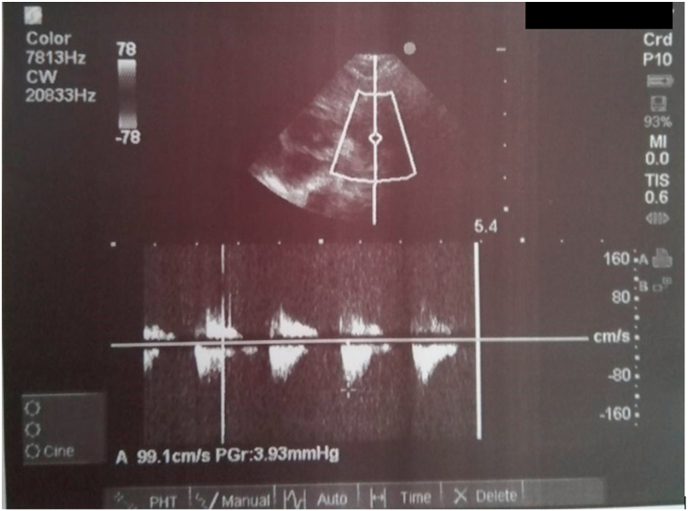
The result of brain ultrasonography, showed choroid plexus 2.7 (mm).

**Fig. 2:**
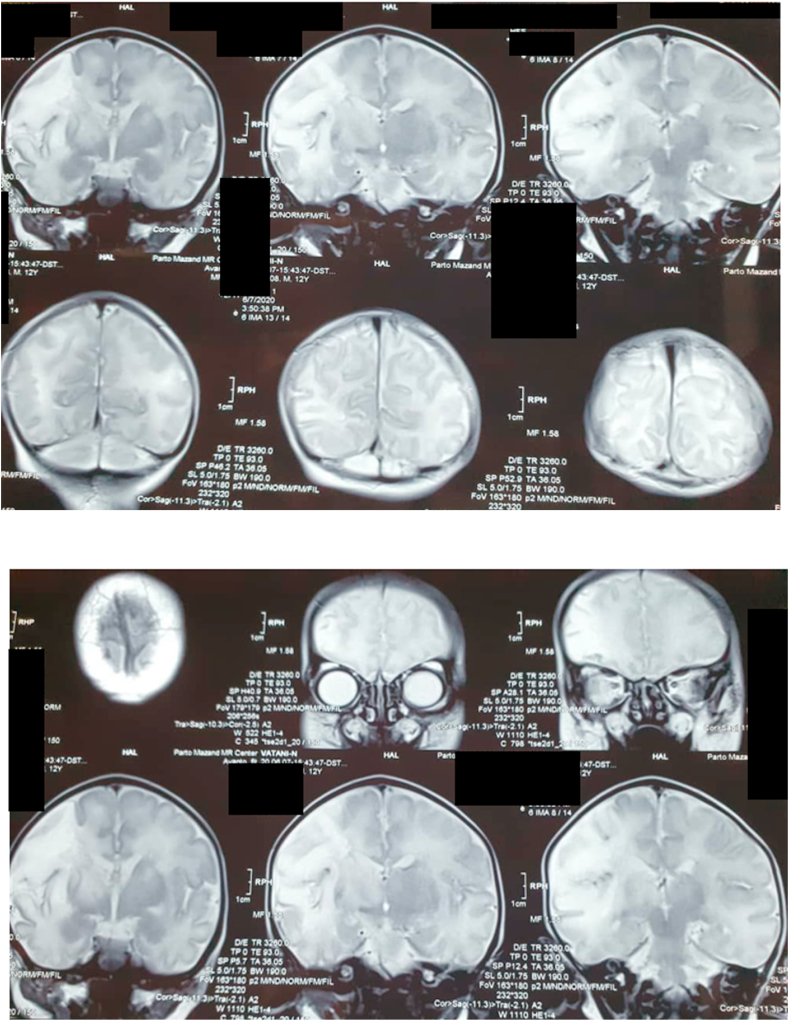
MRI results. Temporoparietal lesion (due to asphyxia) in imaging.

In the COVID-19 quarantine period and due to fear of infection, the mother had delusions of being infected with COVID-19 which caused her to attempt suicide with her medication while she was in the last month of pregnancy and near delivery. However, she survived because of immediate medical intervention. She visited an obstetrician/gynecologist for her pregnancy follow-up and decided to terminate the pregnancy. After delivery, the newborn baby suffered from recurrent seizures and apnea. The physician at the birth center used phenobarbital to treat the newborn's seizure and referred him to our center. The primary lab tests including blood urea nitrogen, creatinine, blood sugar, calcium, total bilirubin, direct bilirubin, potassium, sodium, C-reactive protein, and arterial blood gas (ABG) were requested for the newborn. The test results demonstrated a normal situation except for direct bilirubin and ABG results (Table 1). The increase in the direct bilirubin level was attributed to neonatal icterus, and frequent apnea could have caused ABG abnormality. Therefore, the patient was scheduled for ultrasonography and echocardiography. The results of brain sonography showed 2.7 mm choroid plexus and an absorbing germinal matrix hemorrhage (Fig. 1). Furthermore, abdominopelvic sonography revealed undescended testis and unilateral hydronephrosis (with a diameter of 7/6) in the left kidney; a consultation with a nephrologist and an endocrinologist was requested accordingly. The echocardiography demonstrated atrial septal defect, patent ductus arteriosus, and mild tricuspid regurgitation. Moreover, MRI results confirmed temporoparietal lesions probably due to asphyxia (Fig. 2). After all these examinations, phenytoin, meropenem combined with amikacin, and caffeine were prescribed for his seizure, meconium aspiration, and apnea, respectively. Phenytoin prevented seizures and their recurrence. Nonetheless, five days after phenytoin initiation, dermatologic side effects were observed, including generalized rashes, redness, and maculopapular lesions. Therefore, phenytoin was discontinued and replaced with levetiracetam syrup 0.5 ml twice per day. A dermatologic consultation was requested, followed by prescribing topical hydrocortisone and eucerin. This treatment was effective and rashes subsided in the next five days. Endocrine evaluations represented no pathology, and total thyroxine (T4), free T4, thyroid-stimulating hormone, and blood sugar were in normal ranges. Eventually, the patient was discharged from the hospital after two weeks and scheduled for referral to neurology, nephrology, and cardiac clinics for treating his defects and follow-up. After five months of follow-up, the only sequel left was mild paresthesia of the left upper limb.

**Table 1:** Initial Laboratory data after NICU admission.

Blood culture	Negative
Coombs test direct	Negative
Reticulocyte	1.2%
G.6.P.D	20 U/g Hb
WBC	9000/mm^3^
HCT	45.3%
PLT	225000/mm^3^
HgB	16.2 g/dl
Blood sugar	116 mg/dl
Urea	18 mg/dl
Creatinine	0.6 mg/dl
Calcium	8.4 mg/dl
Bil.T	11 mg/dl
Bil.D	0.9 mg/dl
K	4.1mEq/L
Na	137mEq/L
C.R.P	3 mg/L
Lactate	14 mmol/L
Ammonia	189 μ/dL
TSH	1.8 mIU/L.
T4 Total Thyroxin	6.3 μg/dL
Free T4	14.2 ng/dL
pH	7.341
pCO_2_	34.9 mmHg
Base excess	−6.3 mmol/L
HCO_3_	18.5 mmol/L

## 3 Discussion

Depressive and anxiety disorders are more common in women than men (1.5–2.5 times) whereas schizophrenia is more prevalent in men [11]. Childbirth and pregnancy are introduced as major emotional, physical, and social events in a woman's life. The level of hormones in the blood and its changes are potential causes of altered mental health of women, during pregnancy. Thus, putting women at increased risk of mental health disorders including depression during and after pregnancy [12,13].

Some studies have reported that the COVID-19 pandemic has led to increased levels of anxiety, depression, stress, and alcohol use [14,15]. Women with schizophrenia face a range of adverse pregnancy outcomes including pre-eclampsia, thromboembolic disease, preterm birth, and alterations in fetal growth in comparison with the general population [16]. Recent findings have shown no clinically considerable increase in congenital malformations among the offspring of women taking first- or second-generation antipsychotics during pregnancy. Respiratory distress was also reported in neonates who were exposed to antipsychotic drugs. However, no study has precisely evaluated the relative risk of antipsychotic drugs for fetal growth [17]. For instance, risperidone, which is an atypical antipsychotic drug from benzisoxazole derivatives and was first developed in the 1990s, but there hasn't been sufficient amount of studies on assessment of its safety during pregnancy, so far. Only two studies have reported a case recognized with major organ malformations and four cases with perinatal syndromes (two premature births including one case with a nuchal umbilical cord and a case with a behavioral disorder) as consequences of risperidone use during pregnancy [18].

There have been reports on neonatal malformations due to quetiapine exposures during the first trimester of pregnancy. In animal studies, sertraline has led to the impaired growth of the heart while no report is available regarding significant risk and delivery outcomes following sertraline exposure in human research [19]. Moreover, no evidence exists for increase in congenital malformations in newborns exposed to benzodiazepines such as clonazepam during the first trimester of pregnancy [20]. Also, its effect near delivery remains unclear.

Most studies on drug safety in pregnancy have focused on the long-term effects and therapeutic doses, especially in the first trimester of pregnancy. On the other hand, a limited body of research has investigated the effects of high doses of drugs on suicidal attempts with multi drugs near delivery. This case report suggests that although common psychiatric treatments for patient with psychiatric disorders may have sufficient promising effects, their associated risks and benefits should be assessed before prescription, during the pandemic period. This could extensively impact the patients’ mental health; Highlighting the need for intensive care for patients with psychiatric disorders.

This study was not without limitations. First, Continuous EEG monitoring was not available in our center for the purpose of long-term monitoring. Continuous EEG monitoring is clearly the most reliable technique to detect neonatal seizures, promptly initiate their treatment, and monitor response to treatment.Second, for a more accurate diagnostic examination of the lesions observed in the MRI and to rule out the diagnosis of neonatal stroke, it would be better to perform a Magnetic Resonance Angiography (MRA), which was not available in our center at the time of baby's admission.

## 4 Conclusion

The COVID-19 pandemic has had a serious influence on our lives, lately. The Covid-19 has shown to be associated with exacerbation of some psychiatric disorders, especially in pregnant patients suffering from mental disorders. On the other hand, the safety of use of many antidepressant and antipsychotic drugs during pregnancy remains controversial. Exposure to high doses of antipsychotic drugs could cause suicidal ideation or other side effects in the last months of pregnancy which could eventually lead to fetal harm. However, to date, no study has clearly investigated the safety of these drugs in the last month of pregnancy and near delivery. It is noteworthy that reported neonatal malformations due to risperidone and quetiapine exposures, has created doubts regarding their use during pregnancy. The current case report reinforces the need for further studies to assess the safety of antidepressant and antipsychotic drugs, during pregnancy.

## Ethical Approval

Considering Iran national committee for ethics in biomedical research lows. It's not necessary to get Ethical Approval code for case reports, only patients Consent is enough.

## Sources of funding

The authors didn't used any sources of funding. And this study has no sponsors.

## Author contribution

**J. B,** and **S.K**, Data curation, Writing- Original draft preparation, **SH.M** and **E.P** Reviewing and Editing, **R.F** Supervision.

## Registration of research studies

1. Name of the registry:

2. Unique Identifying number or registration ID:

3. Hyperlink to your specific registration (must be publicly accessible and will be checked):

## Guarantor

Dr. Roya Farhadi, Neonatologist, Associate Professor of Neonatology.

Department of Neonatology, Pediatrics Infectious Diseases Research Center, Faculty of Medicine, Mazandaran University of Medical Sciences, Sari, Iran.

Tel: +98 [11] 33344506.

Email: rfarhadi@mazums.ac.ir.

E-mail: dr.royafarhadi@gmail.com.

## Consent

Written consent for publication of this case report was obtained for publication. A copy of the written consent is available for review by the Editor-in-Chief of this journal on request”.

## Funding sources

This research received no funding.

## Informed consent

Written informed consent was obtained from the patient for publication of this case report and accompanying images. A copy of the written consent is available for review by the Editor-in-Chief of this journal on request.

## Author contributions

**R.F, J. B,** and **S.K** involved in interpretation and collecting of data, and writing- Original draft of the manuscript. **SH.M, E.P** involved in editing the final version of manuscript. All authors reviewed the paper and approved the final version of the manuscript**.**

## Availability of data

The data are available from the corresponding author on reasonable request.

## Provenance and peer review

Not commissioned, externally peer-reviewed.

## Declaration of competing interest

The authors confirm that this article content has no conflict of interest.
